# A Hypothetical Tavern Menu for the Evaluation of Calorie Selection through Menu Labelling

**DOI:** 10.3390/foods11111624

**Published:** 2022-05-31

**Authors:** Katerina Giazitzi, Vasiliki Chrysanthakopoulou, George Boskou

**Affiliations:** Department of Nutrition and Dietetics, School of Health Sciences and Education, Harokopio University, Eleftheriou Venizelou 70, 17676 Athens, Greece; kategiaz@hua.gr (K.G.); dp4422024@hua.gr (V.C.)

**Keywords:** menu, labelling, restaurant, calories, tavern, mark

## Abstract

The purpose of the present study is to evaluate calories selection according to the type of menu labelling applied on a hypothetical Greek tavern menu. Three questionnaires were designed and each one included a hypothetical menu of Greek tavern food. Menu A did not include any nutritional information, Menu B included calorie posting and Menu C had the “NB” mark next to dishes that were Nutritionally Balanced choices. A total of 437 participants were recruited in the study. The mean calories selection was significantly lower on Menu B (1874 Kcal) and C (1787.7 Kcal) compared to Menu A (2157.3 Kcal). The overweight and obese participants ordered significantly fewer calories on Menu B (−504 Kcal and −404 Kcal) and C (−451.3 Kcal and −393 Kcal) than on the Menu A. Menu labelling should be assessed in real-life settings in Greece. It could be a policy for the prevention and control of obesity in European countries.

## 1. Introduction

The prevalence of obesity has risen dramatically in the last forty years, reaching epidemic proportions globally. Obesity is responsible for many non-communicable diseases such as diabetes mellitus, cardiovascular diseases, etc. [1]. It was recently suggested that obesity is also a risk factor for COVID-19 [2]. 

The frequent consumption of meals prepared out-of-home has been associated with increased energy intake and unhealthy eating habits by citizens in southern European countries such as Greece [3,4]. Eating out-of-home can significantly contribute to obesity, as the main cause of it is the overconsumption of energy-dense foods [1].

Various health-related policies have been implemented through the years in order to prevent and control obesity. According to the World Health Organization, menu labelling is one of the strategies to reduce energy intake and control obesity. This typically includes providing precise, understandable and standardized information about the nutritional content of foods on menus in order to raise consumers’ awareness of healthy food choices [5]. On this basis, such policies that encourage healthy food environments, especially in the middle of a pandemic, should be promoted both by the government and the private sector [6].

Menu labelling is a common strategy that aims to empower consumers with information to make healthier choices. In the United States of America (USA), calorie posting is obligatory on the food service outlets that are part of a chain with 20 or more outlets, and certain nutrition information for the menu items are available upon request [7]. Furthermore, a statement indicating the recommended daily calorie intake must be declared. Similarly, in most states in Australia, kilojoule posting and the average daily energy requirements must be displayed on the menus of food service chains [8]. Concerning Europe, there is no united policy about menu labelling with nutritional information. Ireland has moved towards the direction of calorie posting on hospital facilities (canteens etc.) since 2015 [9], and calorie posting has been mandatory since the end of 2019 [10]. In the context of the “Public Health Responsibility Deal”, the United Kingdom (UK) also provided calorie information at food service outlets, mainly until 2015 [11]. 

Various healthy marks and symbols for menus were developed around the world as a helpful tool to effectively inform the consumer. The “Heart Check Mark”, designed by the American Heart Association (AHA), certifies the products, recipes and meals at restaurants that meet specific nutrition requirements based on scientific recommendations of the AHA. This mark is a voluntary labelling scheme. The purpose of the program is to help American citizens choose products suitable for the health of their heart [12]. In Europe, the “Keyhole” logo is a voluntary Nordic mark which is used for both the packaged products and the meals of restaurants and canteens. “Keyhole” has nutritional requirements for the energy, fats, salt, sugars, the content of fruits, vegetables and whole grain products on each menu item [13]. This type of labelling could contribute to the improvement of the nutritional intake for consumers [14]. Moreover, the Traffic Light Labelling (TLL) system was introduced by the UK Food Standards Agency (FSA), and it is a voluntary front-of-pack label of the packaged products. It ranks the products in three colour categories (green, orange or red) according to their level (low, medium or high) of certain nutrients [15]. Many studies positively evaluated its effectiveness on restaurant menus [16,17]. 

Finally, the Physical Activity Calorie Equivalents (PACE) are labels that display the number of minutes or miles of walking (or other forms of exercise) required to burn off the calories provided from one serving of a food item. They are widely used as an alternative type of menu labelling, and especially at order case scenarios. The PACE labels have mixed results as far as their effectiveness in reducing calories ordered [18,19].

The influence of menu labelling on food choices has been widely studied both in real-life settings and in hypothetical orders. Menu labelling has mixed results as far as calorie intake is concerned [20]. A recent meta-analysis resulted in no significant change in reported calories ordered/consumed in natural settings. When the meta-analysis was restricted to studies conducted in laboratory settings, a significant reduction in calories was observed [21]. It has been suggested that the posting of calories with the recommended daily caloric requirements or interpretive nutrition information is the most efficient at limiting total energy intake [22,23]. As far as real menu orders are concerned, calorie posting does not seem to be enough to influence consumers in terms of their meal choice [24]. It is possible that a more descriptive approach (TLLs, PACE, Heart Check Mark, Nutri-score), without verbal information, would be more familiar and understandable to the consumer [18,25,26,27,28,29,30]. As for the hypothetical menu order, the use of traffic-light labelling seems to have mixed results, but mainly it appears to help consumers reduce their calorie intake more than that of calorie posting [30].

Demographic characteristics seem to play an important role in the utilization of nutrition information. More specifically, women are affected more than men by the information that they receive. Women are affected more than men by the provision of nutrition information [31,32,33,34]. Moreover, normal weight consumers were positively influenced by the information compared to overweight/obese consumers [18]. Reale and Flint studied the effect of menu labeling only in obese population. They showed that the participants chose fewer calories on the menu labeling conditions. However, those participants followed a weight management program and they had concerns about their weight. Concerning the menu labeling, the influence of age has not been evaluated before [35].

In Greece, there are only a few efforts at menu labelling on the websites of well-known brands of fast-food chains. The European legislation does not enforce obligatory nutritional information at food services. The purpose of the present study was to assess calories selection when menu labelling exists on a hypothetical Greek tavern menu. Three conditions were evaluated. There was a control menu (no information) and two menus with nutrition information (a. calorie posting and b. Nutritionally Balanced (NB) mark). The menu items which brought the NB mark were designed by a dietitian. It was hypothesized that (1) participants would choose fewer calories on menu-labeling conditions compared to control, (2) they would choose fewer calories at each one of the five menu categories when menu labeling exists, (3) the overweight and obese participants would choose fewer calories than the normal weight ones, (4) women would choose fewer calories on the two menu-labeling conditions, whereas men would not have any difference in the three conditions, (5) the older participants would choose fewer calories on menu-labeling conditions and (6) participants that visit or have visited a dietitian sometimes in their lives or follow a nutritional plan would choose fewer calories on menu-labeling conditions.

## 2. Materials and Methods

### 2.1. Data Collection

For the purpose of this study, three questionnaires were designed based on the previous study of Liu et al. (2012) [26]. The methodology of this previous study was implemented because the researchers wanted to evaluate the possible nutritional intake. They did not evaluate nutritional behavior. The variables under consideration are all numerical. The questionnaires were tested upon 20 volunteers and by a dietitian who conducted interviews in order to check the clarity of the context and the functionality of evaluation.

The hypothetical menu orders were designed on internet-based forms with the Google forms application. Participants were randomly recruited to take part in the study with posts on social media. The social media network was used in order to ensure the safety of the researchers and the participants, due to the COVID-19 pandemic. Every participant provided informed consent on the very first page of the questionnaire. The study was approved by the Institutional Ethics Review Board of the Harokopio University under protocol number 423/11.02.2020.

The collection of data lasted for four months, between February 2020 to July 2020. Overall, 437 participants successfully completed the three phases of the study. All three phases (conditions) consist of an e-menu for a hypothetical Greek tavern. Firstly, the supposed customers provided information about demographics, frequency of eating out-of-home and if they follow certain nutritional advice or a weight management program. Then, a transitional page appeared with a message that motivated the participants to choose the menu items they would order if they were in a traditional Greek tavern/restaurant at the time of completing the survey. A hypothetical Greek restaurant menu appeared and each participant could virtually order what he wanted to eat, with a limit of expenditure up to 25 euros. The menu was based on a typical Greek traditional tavern with 5 menu categories [36]. These categories were appetizers (8 menu items), salads (7 menu items), main dishes (19 menu items), desserts (7 menu items) and beverages (all the possible items that a tavern can offer). The price of each portion was displayed to the right of each menu item. Regarding salads, appetizers and beverages, participants had the option to choose between half, one, two or three portions of each menu item. This possibility is also plausible in a traditional Greek restaurant/tavern, where typical appetizers or salads are usually for 2 to 4 people.

After a washout period of 15–25 days, participants received an e-mail with a link to the second hypothetical menu. The procedure was repeated once more. The difference between the three hypothetical menus was the type of nutritional information displayed on each one. Menu A did not contain any nutritional information (control), Menu B listed the calories per serving to the right of each menu item (before the price) (calorie posting) and Menu C had a distinctive mark (Nutritionally Balanced mark) to the right of the items (before the price) that were balanced options (per serving). In particular, the mark was the Greek symbol of Delta, which is also the first letter of the word diet and nutrition in the Greek language. The NB menu items were based on recipes of traditional Greek dishes, but they were designed and adjusted to the criteria by a dietitian. The participants were informed about the contribution of the dietitian to the menu. It should be mentioned that a menu item could obtain the Nutritionally Balanced (NB) mark if it met specific nutritional limits. The nutritional limits were about the energy content of the dishes. The NB salads contained up to 250 Kcal/serving, the NB appetizers up to 250 Kcal/serving, the NB main dishes up to 600 Kcal/serving and the NB desserts up to 200 Kcal/serving [37,38]. No particular limits were set for the beverages. 

The hunger level prior to study and the fasting hours of the participants were evaluated in relation to each one of the three menu options. The hunger level was rated on a 7-point Likert scale (“not at all” to “very much”). The importance of price, taste, calories (on Menu B) and NB mark (on Menu C) on ordering was also evaluated on a 7-point Likert scale (“not at all” to “very much”).

The participants who completed all three phases (conditions) of the study received an email of gratitude with an e-cookbook attached, containing Nutritionally Balanced recipes edited by the research team.

In total, 550 participants were recruited for the study. The second phase (condition) was completed by 480 people, while 470 people went through the third phase. However, only 438 people were identified with their email addresses to answer all three questionnaires. One person was excluded from the sample, as he selected an extreme number of menu items and total calories in all three phases of the study (approximately 15,000 calories). Thus, the total number of participants in the study was 437 people.

### 2.2. Statistical Analysis

Data analysis was performed with SPSS version 21.0. Descriptive statistics were performed to summarize the continuous variables. Data did not follow normal distribution according to Kolmogorov–Smirnov tests (*p*-value < 0.05). After exploring variable distributions, the Wilcoxon signed ranks test was used to identify differences among menus concerning hunger level, the hours since the last meal prior to study, the importance of price and taste and nutritional value of the selected dishes, with statistical significance set at *p*-value < 0.05. Pearson correlations were performed between the overall selected calories and the calories from the menu categories in order to identify which category contributes more to the total selected categories, with *p*-value level < 0.001. The Wilcoxon signed ranks test was performed to assess differences in calories among each menu category for the three different menus and among the four BMI categories. The Kruskal–Wallis ANOVA analysis and post hoc analysis were used to evaluate calorie differences between participants who were trying to lose weight or control/reduce the calories they consume and those who were not. Statistical significance was set at *p*-value ≤ 0.05. The same analyses were conducted for each one of the four BMI categories in order to identify the type of labelling that had the greatest influence on selected calories, with statistical significance at *p*-value ≤ 0.001.

## 3. Results

The majority of the participants were women (72.8%). Table 1 presents a summary of the demographic characteristics and of the responses of several ordinal variables. There are no differences among menus concerning hunger level and the hours since the last meal prior to study (*p* > 0.01).

The sample contained 208 (47.6%) participants that had visited a dietitian/nutritionist at some time in their lives. The 51% of the responders followed a nutrition plan during the study, and 133 persons had taken nutritional advice from a health care provider sometime during their lives. The majority of the responders (59.7%) had normal Body Mass Index (BMI), 22.7% were overweight and 14.6% were obese. 

### 3.1. Selected Calories per Menu Category

**Figure 1:**
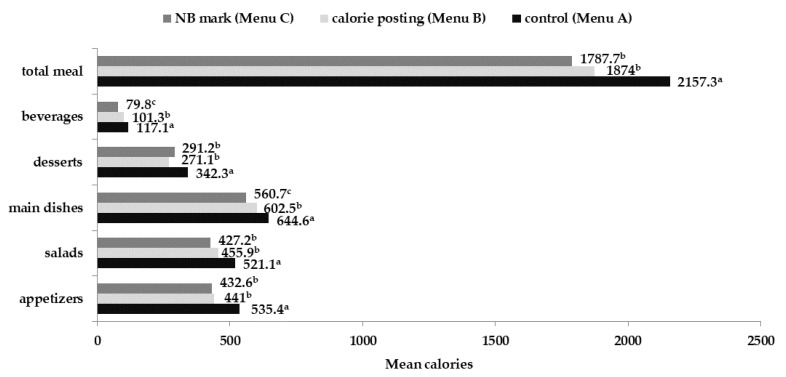
Mean selected calories for each menu category for the three different menus. The superscript letters (a,b,c) display the statistically significant differences (*p* < 0.05) between the selected calories per menu category (appetizer, salads etc.).

Figure 1 presents the mean selected calories per order for each menu category. Wilcoxon signed ranks test and post hoc analysis showed that there are statistically significant differences between Menu A and the two other menus as far as appetizers, salads and desserts are concerned. Participants ordered fewer calories from appetizers on Menu B (calorie posting) (−94.4 Kcal) and C (NB mark) (−102.8 Kcal) compared to Menu A (*p* < 0.001). Similarly, they ordered fewer calories from salads on Menu B (−65.2 Kcal) (*p* = 0.002) and C (−93.9 Kcal) compared to Menu A (*p* < 0.001). Moreover, responders selected fewer calories from desserts on Menu B (−71.2 Kcal) (*p* < 0.001) and C (−51.1 Kcal) compared to Menu A (*p* < 0.001). The mean selected calories on the main dishes of Menu B were 36.8 kcal fewer than the calories on Menu A (*p* = 0.009). Participants also chose 84.4 fewer calories from main dishes on Menu C than on Menu A (*p* < 0.001). In addition, they selected 47.6 more calories from the main dishes of Menu B compared to the calories selected from the main dishes of menu C (*p* = 0.045). Participants chose fewer calories from drinks in Menu B (−15.8 Kcal) (*p* < 0.05) and C (−37.3 Kcal) (*p* < 0.001) compared to their choice in Menu A. The overall selected calories per order were significantly less on Menu B (−283.3 Kcal) and C (−369.6 Kcal) compared to Menu A (*p* < 0.001) (Figure 1).

The Pearson correlations were performed between the overall selected calories and the calories from the subcategories of the menus (appetizers, salads, main dishes, desserts and beverages). The total selected calories on menu A (2157.3 Kcal), B (1874 Kcal) and C (1787.7 Kcal) are quite well correlated with appetizers (R_menuA_ = 0.782, R_menuB_ = 0.747, R_menuC_ = 0.716) (*p* < 0.001). The salads (R_menuA_ = 0.593, R_menuB_ = 0.530, R_menuC_ = 0.554), main dishes (R = 0.442, R_menuB_ = 0.535, R_menuC_ = 0.513), desserts (R_menuA_ = 0.442, R_menuB_ = 0.402, R_menuC_ = 0.462) and beverages (R = 0.324, R_menuB_ = 0.369, R_menuC_ = 0.294) have a weak correlation with the total calories for all of the three menu conditions (*p* < 0.001). 

### 3.2. Selected Calories per BMI Category

**Figure 2:**
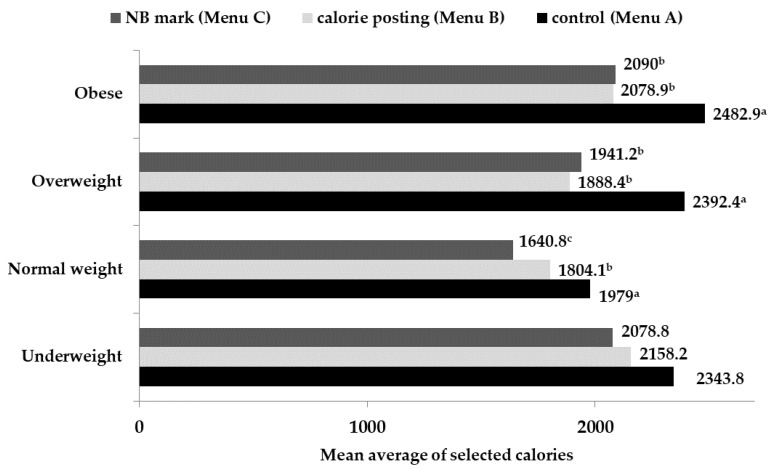
Mean selected calories among the four BMI categories for each menu (NB mark, control, calorie posting). The superscript letters (a,b,c) display the statistically significant differences between the selected calories on each menu per BMI category (*p* ≤ 0.001).

A Wilcoxon signed ranks test was conducted for each of the four BMI categories. The normal weight participants chose fewer calories on Menu B (−174.9 Kcal) and Menu C (−338.1 Kcal) compared to Menu A (*p* ≤ 0.001). The mean difference between Menu B and Menu C for the normal weight participants was 163.3 Kcal (*p* = 0.001). The overweight participants ordered significantly fewer calories on the menu with calorie posting (−504 Kcal) and NB mark (−451.3 Kcal) than on the menu without information (*p* < 0.001). The ordered calories on Menu B and C had no differences. Similarly, the obese responders did not order a different number of calories on Menu B and C. However, they chose fewer calories on Menu B (−404 Kcal) and C (−393 Kcal) than on Menu A (*p* ≤ 0.001) (Figure 2).

The mean average of selected calories per order was evaluated for each BMI category. According to Kruskal–Wallis ANOVA analysis and post hoc analysis, overweight (+413.4 Kcal) (*p* = 0.003) and obese (+503.9 Kcal) (*p* = 0.002) participants chose significantly more calories than the normal weight ones on Menu A. The selected calories on Menu B did not differ between the BMI categories. In the case of Menu C, the normal weight participants selected fewer calories than the overweight (+300.4 Kcal) (*p* < 0.02) and obese (+449.2 Kcal) persons (*p* = 0.001).

### 3.3. Other Variables

Concerning women, they chose significantly fewer calories on the calorie-posting condition (−308.4 Kcal) (*p* < 0.001) and on the NB-mark condition (−380.8 Kcal) (*p* < 0.001) compared to the menu without information (2040.7 Kcal). Men followed the same pattern, as they chose 2469 Kcal on the menu without information, 2252.6 Kcal on the menu with calorie posting (−216.4 Kcal) (*p* < 0.02) and 2129.3 Kcal on the menu with the NB mark (−339.7 Kcal) (*p* < 0.001). No differences were detected among the age groups as far as selected calories. Moreover, there are no differences between the participants that had visited a dietitian/nutritionist or had consulted a health care professional (doctor etc.) sometime in their lives and those that had not taken advice from anyone (*p* > 0.05).

It turns out that 51% of the hypothetical customers followed a nutrition plan during the study (at least they declared so). The remaining 49% of the participants did not follow a plan or they were not sure. Table 2 presents the Kruskal–Wallis ANOVA analysis for the question, “Are you currently trying to lose weight or control/reduce the calories you consume?” Participants who were trying to lose weight statistically selected the same calories as participants who were not trying to lose weight (*p* > 0.05). However, those who were in a weight loss program rated the importance of the nutritional value of the selected dishes higher than the rest of the participants (*p* < 0.05) (Table 2).

## 4. Discussion

This is the first study to evaluate the different-ordered-calories-per-menu labelling condition in the Greek population. The menu was a typical one for a Greek sit-down restaurant (tavern). Moreover, it is the first study to introduce dishes designed by a professional dietitian. Participants chose fewer calories on the two menu-labelling conditions. Previous studies have evaluated the influence of menu labelling on hypothetical orders for well-known fast-food outlets (such as McDonalds and Burger King) [18,19,30] or American style chain restaurants (such as Applebee’s) [26] or e-canteens with sandwiches made from scratch [39]. These studies were conducted in USA, UK and Australia. No other similar study has been conducted in Europe or especially in Greece. The present one is considered original, as no one else has studied the Greek population as far as menu labelling is concerned. The populations of the USA, Australia and UK are familiar with the calorie/kilojoule posting on menus, in contrast with the Greek population. This policy of calorie posting is voluntary for food services in the UK [11] and obligatory for Ireland [10], USA [7] and Australia (food service chains with more than 20 outlets) [8]. This was the first interaction of the Greek participants with menu labelling. They only experience the nutritional labelling of packaged products, though it has not been clarified yet if they understand or use this information.

### 4.1. Interpretation of Findings and Comparison with Previous Studies

The calorie posting and NB mark influenced the hypothetical orders, as selected calories were lower on these two conditions compared to control menu. In particular, the selected calories from appetizers, salads and desserts were less on the calorie posting and the NB meals than on the menu with no information. The participants chose the fewest calories from the main dishes and the beverages on the menu with the NB marks compared to the two other conditions. In contrast, Lee & Thompson (2016) showed that the selected calories from the side dishes were lower on no information cases and the “calorie plus walking miles” labelling than on the simple calorie-posting menu, whereas participants selected higher calories from beverages in “calories plus walking miles” labelling than on the simple calorie-posting menu. They also presented no differences on main dishes (burgers) among the three menu conditions (a. no information, b. calorie posting, c. calorie posting and miles to walk to burn those calories) [19].

In total, participants chose approximately 325 Kcal (283.3–369.6 Kcal) less energy on the two menu-labelling conditions than on the menu without labelling. Apart from the statistical figures, these calories also have clinical significance for metabolic energy balance. In particular, Wishnofsky (1958) showed that a negative energy balance of 3500 Kcal can lead to a weight loss of 0.45 Kg [40]. According to the present study, the participants eat out-of-home meals approximately two times per week. That means that they can achieve a loss or a prevention of weight gain of approximately 4 Kg per year, without any other change in their nutrition and exercise habits.

The impact of menu labelling is evaluated across BMI categories. The normal weight participants chose the least number of calories on the menu with the NB meals. Indeed, the mean difference of calories between the menu without information and the menu with the NB mark was about 338.2 Kcal. Overweight and obese participants had a positive effect from both calorie posting and NB marked menus. They chose between 393–504 fewer calories than the control situation. This mean difference is in agreement with Reale and Flint (2016), who showed a decrease of 317.2 Kcal between control and the calorie-posting condition on the overweight/obese population [35].

The present study indicated that participants who followed a nutrition plan for weight management chose fewer calories on the calorie-posting condition. This result is in accordance with Reale and Flint, who showed that the participants who had joined a weight management program were motivated to eat healthily and to lose weight [35]. At the same time, a previous study showed that consumers who focus on the healthfulness of foods choose fewer calories than those that focus on the quantity [41]. Their motivation as well as their possible higher nutrition literacy may play a key role on the quality of the chosen meals [42]. 

In contrast, another study showed that menu labelling did not influence the calorie choice of overweight/obese volunteers. Researchers had one control group (no labelling) and three intervention groups (a. calorie posting, b. calorie posting and “minutes to walk to burn those calories” and c. calorie posting and “miles to walk to burn those calories”). They involved underweight/normal weight and overweight and obese participants, and they indicated that only underweight/normal weight ones were affected by the menu-labelling conditions compared to the no-labelling menu. The intervention menu, with the calories and “the miles to walk to burn those calories”, had the greatest impact on the selected calories for underweight/normal weight participants [18].

Both men and women chose fewer calories on the calorie-posting condition and on NB-mark menu, compared to the control menu. Previous studies [31,34] compared men to women, and they showed that women chose fewer calories than the men on menu labeling conditions. This is expected, as women have lower nutritional and energy requirements than the men. At the same time, a study has shown that women are more aware of the healthfulness of their food [43]. As far as age groups are concerned, the present study did not find any difference among them. However, Ellison et al. showed that younger consumers were more likely to order medium- to high-calorie entrées, in contrast with older participants, who tended to choose low-calorie entrées, when menu labeling is available [34].

The overall selected calories per order are considered high (1787.7–2157.3 Kcal) for a single meal. Liu et al. (2012) presented also an increased choice of calories (1454.55–1759.61 Kcal) on the four menu conditions, but the calories ordered on the menu without labelling was close to the selected calories of the menu with the NB meals of our study [26]. Another research study evaluated the PACE menu labelling and it presented a high-calorie choice of 1140–1580 Kcal according to one of the four menu conditions (similar menu conditions with Dowray et al., 2013) [44]. It is important to mention that the hypothetical menu orders can evaluate the intention to order and to eat a meal but not the actual calorie intake. Therefore, these calories might be lower in real-life settings, or, sometimes, even more. 

A statistically significant impact of calorie posting and the NB mark was observed on selected calories, compared to the no-information condition. The menu with the NB meals has the greatest impact on the normal weight persons, whereas overweight and obese persons are affected by any type of nutrition information on the menu. Furthermore, both men and women are influenced more by the menu with the NB meals. According to other researchers, the calorie posting (numerical/quantitative information) on menus has several disadvantages, as it demands numerical skills and time to process the information [45]. However, customers have no particular time constraints in a sit-down restaurant (such as a Greek tavern), as they have in a fast-food outlet. Therefore, they can better process numerical information [26].

### 4.2. Strengthens and Weaknesses

Some of the advantages of this study are the large sample size and the constant hunger level prior to study on the three conditions. Moreover, the price, the dishes and the serving sizes were constant across menu conditions. The evaluation of a Greek tavern menu differentiates the present work from the previous studies with hypothetical menu orders. The present study has several limitations. The hypothetical nature of the menu is a limitation, as the actual food and calorie intake is not evaluated. In the hypothetical orders, it is not possible to assess how much they would not eat if they were actually in a restaurant. This can only be evaluated in real-life settings. The environmental factors (odours, colours, noises) and social pressure may influence the final choice of dishes and food intake in a food service outlet [46,47]. The number of men was only 30% of the overall participants. However, it was observed that women are more aware of their nutrition compared to men [43]. Therefore, it was expected that there would be a small recruitment of them in the study. In addition, participants reported their own weight and height. An underestimation of true BMI may have occurred [48]. The constant dishes through the three conditions are a strength and limitation at the same time. Even though there was a washout period of 15–25 days, the repeated exposure may have influenced the choices of the participants, mainly on the final menu with the NB meals. 

### 4.3. Implications for Further Research and Practice

This study enhances the existing literature by examining the effect of the new approach of Nutritionally Balanced (NB) options on a menu, which have been designed by a dietitian. The effect of the NB marking compared with that of the calorie posting and the control (no information) is quite prominent. The approach of the NB-marked dishes on the menu enables participants to distinguish at a glance which ones are healthy based on specific nutritional criteria. Therefore, the consumers can make a healthy choice when eating out-of-home, even though they have no knowledge or education regarding healthy eating. Indeed, Traffic Light Labelling [30], ranking of the dishes as displayed on menu from lower to higher energy content and vice versa [26], presentation of Health Statements [17], PACE equivalents [18,19] and presentation of the relative differences in calories among the food items (for example relative to the food item with the lowest calories) [39] have better results on the calories ordered compared to a calorie-posting label.

The use of marks/symbols (like NB mark) is a kind of a “nudge” that affects customer choices without forbidding any ordering options. According to Thaler and Sunstein, who first introduced the concept of “nudge”, it is small changes in environmental architecture that differentiate the consumers’ decisions and choices without forbidding any other option [49]. There are also other aspects that should be investigated in future research of populations without experience on menu labelling. For instance, the presentation of calories prior to the menu item (on the left) [50] and the presentation of the healthier food items first on the menu list are considered to lead to less calories ordered and healthier choices [51]. Eye-tracking is an emerging methodology to monitor the visual attention of the customers concerning the menu labelling [52]. The evaluation of health and nutrition literacy in the interpretation of the two menu labels (calorie posting and NB mark) could provide new evidence for the selected meals and calorie intake [23].

The impact and effectiveness of menu labelling (calorie posting or NB mark) should be evaluated in real-world settings in Greece (for example in a Greek tavern/restaurant). The pilot implementation of menu labelling could be feasible in a food service outlet in Greece, as many of them use e-menus, due to the COVID-19 health crisis [6]. After the end of this crisis, the evaluation of the actual food and calorie intake could be conducted in a study with real orders using the method of visual plate waste [53].

## 5. Conclusions

The results for the potential Greek consumers were similar to those for other countries, even though they had not experienced previous initiatives with menu labelling. Both Greek and other European populations could benefit from an initiative like this. The introduction of the NB options on menus could lead to the selection of fewer calories on menu orders. As similar findings have emerged from studies in other continents, it arises that mandatory menu labelling could be part of a comprehensive action to reduce the prevalence of overweight and obesity. In the context of legislation, the education of the consumers about menu labelling would be necessary to use the provided information to their advantage.

## Figures and Tables

**Table 1:** Descriptive statistics for the numerical and ordinal variables (The * indicates statistically significant differences according to the Wilcoxon signed ranks test, with statistical significance set at *p* < 0.05).

	Min–Max	Mean (SE)	SD	
Age (years)	18–67	34.5 (0.6)	12.04	
Height (m)	1.50–2.00	1.68 (0.004)	0.09	
Weight (kg)	43–140	70.1 (0.8)	16.8	
BMI (kg/m^2^)	15.6–43.4	24.6 (0.2)	5	
Frequency of eating out-of-home (times per week)	0–8	1.9 (0.08)	1.7	
*p*-value				
Hours since the last meal				
(Menu A)	0–72	3.3 (0.25)	5.3	<0.05
(Menu B)	0–19	3.1 (0.15)	3.1	
(Menu C)	0–40	2.7 * (0.16)	3.3	
Hunger level prior to study				
(Menu A)	1–7	2.4 (0.07)	1.5	>0.05
(Menu B)	1–7	2.5 (0.08)	1.6	
(Menu C)	1–7	2.5 (0.08)	1.7	
How important was the price for choosing each dish?				
(Menu A)	1–7	3.4 * (0.08)	1.6	<0.001
(Menu B)	1–7	3 (0.08)	1.6	
(Menu C)	1–7	2.9 (0.08)	1.6	
How important was the taste for choosing each dish?				
(Menu A)	2–7	5.9 (0.06)	1.3	>0.05
(Menu B)	1–7	6 (0.05)	1.1	
(Menu C)	1–7	6 (0.05)	1.1	
How important was the nutritional value for choosing each dish?				
(Menu B)	1–7	3.5 (0.08)	1.7	<0.001
(Menu C)	1–7	4.4 (0.08)	1.6	

**Table 2:** Kruskal–Wallis ANOVA analysis. The superscript letters (^a,b,^ display the statistically significant differences, with statistical significance set at *p* level < 0.05.

		Are You Currently Trying to Lose Weight or Control/Reduce the Calories You Consume?			
		Yes (n = 223)	No (n = 175)	I Am Not Sure (n = 39)	*p*
Mean (SD)	selected calories on menu without information	2198.4 ^a^ (1147.9)	2007.7 ^a^ (810.4)	2593.6 ^b^ (1008)	<0.05
	selected calories on calorie posting menu	1805.1 ^a^ (930)	1864.3 ^a^ (860.3)	2311.7 ^b^ (884)	<0.05
	selected calories on menu with the NB mark	1797.6 ^a,b^ (850.4)	1712.8 ^a^ (827.1)	2066.8 ^b^ (776.3)	<0.05
	How important was the nutritional value for you when choosing the dish on calorie posting menu **	3.98 ^a^ (1.65)	2.98 ^b^ (1.65)	3.15 ^b^ (1.4)	<0.05
	How important was the nutritional value for you when choosing the dish on menu with the NB mark **	4.63 ^a^ (1.52)	4.13 ^b^ (1.81)	3.9 ^b^ (1.23)	<0.05

** The importance of the nutritional value is measured on a scale of 1–7.

## References

[1] World Health Organization. https://www.who.int/news-room/fact-sheets/detail/obesity-and-overweight.

[2] Kassir R. (2020). Risk of COVID-19 for patients with obesity. Obes. Rev..

[3] Orfanos P., Naska A., Trichopoulos D., Slimani N., Ferrari P., Van Bakel M., Deharveng G., Overvad K., Tjønneland A., Halkjær J. (2007). Eating out of home and its correlates in 10 European countries. The European Prospective Investigation into Cancer and Nutrition (EPIC) study. Public Health Nutr..

[4] Orfanos P., Naska A., Trichopoulou A., Grioni S., Boer J.M.A., Van Bakel M.M.E., Ericson U., Rohrmann S., Boeing H., Rodríguez L. (2009). Eating out of home: Energy, macro-and micronutrient intakes in 10 European countries. The European Prospective Investigation into Cancer and Nutrition. Eur. J. Clin. Nutr..

[5] World Health Organization. http://www.who.int/mediacentre/factsheets/fs394/en/.

[6] Rincón-Gallardo P.S., Zhou M., Da Silva Gomes F., Lemaire R., Hedrick V., Serrano E., Kraak V.I. (2020). Effects of Menu Labeling Policies on Transnational Restaurant Chains to Promote a Healthy Diet: A Scoping Review to Inform Policy and Research. Nutrients.

[7] Food and Drug Administration U.S. Department of Health and Human Services Center for Food Safety and Applied Nutrition. https://www.fda.gov/regulatory-information/search-fda-guidance-documents/guidance-industry-menu-labeling-supplemental-guidance.

[8] Obesity Policy Coalition. https://www.opc.org.au/downloads/policy-briefs/menu-kj-labelling-in-chain-food-outlets-in-australia.pdf.

[9] Health Service Executive. https://www.hse.ie/eng/about/who/healthwellbeing/our-priority-programmes/heal/calorie-posting/.

[10] Fitzpatrick P., Flood C., Cuniffe E., Doherty K., Lyons A., Stynes S., Pilkington A., Barnes L., Peare T., Kelleher C.C. (2019). Learning from calorie posting/traffic light systems introduction in a University hospital canteen. Eur. J. Public Health.

[11] British Nutrition Foundation. https://www.nutrition.org.uk/nutritioninthenews/reports/responsibility-deal.html.

[12] American Heart Association. https://www.heart.org/en/healthy-living/company-collaboration/heart-check-certification/why-you-can-trust-the-heart-check-mark.

[13] Danish Veterinary and Food Administration (2015). Facts, Report No.: Fact-Book-DVA-2015.

[14] Lassen A.D., Beck D.A., Leedo E., Andersen E.W., Christensen T., Mejborn H., Thorsen A.V., Tetens I. (2014). Effectiveness of offering healthy labelled meals in improving the nutritional quality of lunch meals eaten in a worksite canteen. Appetite.

[15] Department of Health and Social Care. https://www.gov.uk/government/publications/front-of-pack-nutrition-labelling-guidance.

[16] Bleich S.N., Economos C.D., Spiker M.L., Vercammen K.A., VanEpps E.M., Block J.P., Elbel B., Story M., Roberto C.A.A. (2017). A systematic review of calorie labeling and modified calorie labeling interventions: Impact on consumer and restaurant behaviour. Obesity.

[17] Pang J., Hammond D. (2013). Efficacy and consumer preferences for different approaches to calorie labeling on menus. J. Nutr. Educ. Behav..

[18] Dowray S., Swartz J.J., Braxton D., Viera A.J. (2013). Potential effect of physical activity based menu labels on the calorie content of selected fast food meals. Appetite.

[19] Lee M.S., Thompson J.K. (2016). Exploring enhanced menu labels’ influence on fast food selections and exercise-related attitudes, perceptions, and intentions. Appetite.

[20] VanEpps E.M., Roberto C.A., Park S., Economos C.D., Bleich S.N. (2016). Restaurant menu labeling policy: Review of evidence and controversies. Curr. Obes. Rep..

[21] Cantu-Jungles T.M., McCormack L.A., Slaven J.E., Slebodnik M., Eicher-Miller H.A. (2017). A meta-analysis to determine the impact of restaurant menu labeling on calories and nutrients (ordered or consumed) in US adults. Nutrients.

[22] Roberto C.A., Larsen P.D., Agnew H., Baik J., Brownell K.D. (2010). Evaluating the impact of menu labeling on food choices and intake. Am. J. Public Health.

[23] Sinclair S.E., Cooper M., Mansfield E.D. (2014). The influence of menu labeling on calories selected or consumed: A systematic review and meta-analysis. J. Acad. Nutr. Diet..

[24] Kiszko K.M., Martinez O.D., Abrams C., Elbel B. (2014). The influence of calorie labeling on food orders and consumption: A review of the literature. J. Community Health.

[25] McGuire S. (2012). Institute of Medicine. Front-of-Package Nutrition Rating Systems and Symbols: Promoting Healthier Choices. Washington, DC: The National Academies Press. Adv. Nutr..

[26] Liu P.J., Roberto C.A., Liu L.J., Brownell K.D. (2012). A test of different menu labeling presentations. Appetite.

[27] Vanderlee L., Hammond D. (2013). Does nutrition information on menus impact food choices? Comparisons across two hospital cafeterias. Public Health Nutr..

[28] Bleich S.N., Herring B.J., Flagg D.D., Gary-Webb T.L. (2012). Reduction in purchases of sugar-sweetened beverages among low-income black adolescents after exposure to caloric information. Am. J. Public Health.

[29] Levy D.E., Riis J., Sonnenberg L.M., Barraclough S.J., Thorndike A.N. (2012). Food choices of minority and low-income employees: A cafeteria intervention. Am. J. Prev. Med..

[30] Morley B., Scully M., Martin J., Niven P., Dixon H., Wakefield M. (2013). What types of nutrition menu labelling lead consumers to select less energy-dense fast food? An experimental study. Appetite.

[31] Dumanovsky T., Huang C.Y., Nonas C.A., Matte T.D., Bassett M.T., Silver L.D. (2011). Changes in energy content of lunchtime purchases from fast food restaurants after introduction of calorie labelling: Cross sectional customer surveys. BMJ.

[32] Gerend M. (2009). Does calorie information promote lower calorie fast food choices among college students?. J. Adolesc. Health.

[33] Krieger J.W., Chan N.L., Saelens B.E., Ta M.L., Solet D., Fleming D.W. (2013). Menu labeling regulations and calories purchased at chain restaurants. Am. J. Prev. Med..

[34] Ellison B., Lusk J.L., Davis D. (2013). Looking at the label and beyond: The effects of calorie labels, health consciousness, and demographics on caloric intake in restaurants. Int. J. Behav. Nutr. Phys. Act..

[35] Reale S., Flint S.W. (2016). Menu labelling and food choice in obese adults: A feasibility study. BMC Obes..

[36] Palisidis G., Boskou G., Stavridi E. (2013). Strategy in Traditional Restaurants. Analysis and Evaluation of menus at food service outlets. J. Tour. Res..

[37] American Heart Association. https://www.heart.org/en/healthy-living/company-collaboration/heart-check-certification/heart-check-certified-recipes/heart-check-recipe-certification-program-nutrition-requirements.

[38] Giazitzi K., Boskou G. (2021). Developing a methodology to create nutritionally balanced meals. Br. Food J..

[39] Gustafson C.R., Zeballos E. (2020). The effect of presenting relative calorie information on calories ordered. Appetite.

[40] Wishnofsky M. (1958). Caloric equivalents of gained or lost weight. Am. J. Clin. Nutr..

[41] Berry C., Burton S., Howlett E., Newman C.L. (2019). Understanding the calorie labeling paradox in chain restaurants: Why menu calorie labeling alone may not affect average calories ordered. J. Public Policy Mark..

[42] Gibbs H.D., Ellerbeck E.F., Gajewski B., Zhang C., Sullivan D.K. (2018). The nutrition literacy assessment instrument is a valid and reliable measure of nutrition literacy in adults with chronic disease. J. Nutr. Educ. Behav..

[43] Glanz K., Basil M., Maibach E., Goldberg J., Snyder D.A.N. (1998). Why Americans eat what they do: Taste, nutrition, cost, convenience, and weight control concerns as influences on food consumption. J. Am. Diet. Assoc..

[44] Antonelli R., Viera A.J. (2015). Potential effect of physical activity calorie equivalent (PACE) labeling on adult fast food ordering and exercise. PLoS ONE.

[45] Hoefkens C., Lachat C., Kolsteren P., Van Camp J., Verbeke W. (2011). Posting point-of-purchase nutrition information in university canteens does not influence meal choice and nutrient intake. Am. J. Clin. Nutr..

[46] Clauzel A., Guichard N., Riché C. (2019). Dining alone or together? The effect of group size on the service customer experience. J. Retail. Consum. Serv..

[47] Spence C., Meiselman H. (2020). Atmospheric Effects on Eating and Drinking: A Review. Handbook of Eating and Drinking.

[48] Rowland M.L. (1990). Self-reported weight and height. Am. J. Clin. Nutr..

[49] Thaler R.H., Sunstein C.R. (2008). Nudge: Improving Decisions about Health, Wealth, and Happiness.

[50] Dallas S.K., Liu P.J., Ubel P.A. (2019). Don’t count calorie labeling out: Calorie counts on the left side of menu items lead to lower calorie food choices. J. Consum. Psychol..

[51] Policastro P., Smith Z., Chapman G. (2017). Put the healthy item first: Order of ingredient listing influences consumer selection. J. Health Psychol..

[52] Schwebler S.A., Harrington R.J., Ottenbacher M.C. (2020). Calorie disclosure and color-coding on QSR menus: A multi-method approach using eye-tracking technology, grouping and surveys. Int. J. Hosp. Tour. Adm..

[53] Connors P.L., Rozell S.B. (2004). Using a visual plate waste study to monitor menu performance. J. Am. Diet. Assoc..

